# Trajectory analysis reveals an uncommitted neuroblastic state in MYCN-driven neuroblastoma development

**DOI:** 10.1093/neuonc/noaf129

**Published:** 2025-06-24

**Authors:** Shoma Tsubota, Daniel R Carter, Janith A Seneviratne, Haruka Hirose, Teppei Shimamura, Yukie Kashima, Yutaka Suzuki, Koji Tsuda, Glenn M Marshall, Kenji Kadomatsu

**Affiliations:** Department of Biochemistry, Nagoya University Graduate School of Medicine, Nagoya, Japan; School of Biomedical Engineering, University of Technology Sydney, Sydney, NSW, Australia; Children’s Cancer Institute Australia for Medical Research, Lowy Cancer Research Centre, UNSW Sydney, Kensington, NSW, Australia; Children’s Cancer Institute Australia for Medical Research, Lowy Cancer Research Centre, UNSW Sydney, Kensington, NSW, Australia; Division of Systems Biology, Nagoya University Graduate School of Medicine, Nagoya, Japan; Division of Systems Biology, Nagoya University Graduate School of Medicine, Nagoya, Japan; Department of Computational Biology and Medical Sciences, Graduate School of Frontier Sciences, The University of Tokyo, Kashiwa, Japan; Department of Computational Biology and Medical Sciences, Graduate School of Frontier Sciences, The University of Tokyo, Kashiwa, Japan; Department of Computational Biology and Medical Sciences, Graduate School of Frontier Sciences, The University of Tokyo, Kashiwa, Japan; Kids Cancer Centre, Sydney Children’s Hospital, Randwick, NSW, Australia; School of Clinical Medicine, UNSW Medicine & Health, UNSW Sydney, Sydney, NSW, Australia; Children’s Cancer Institute Australia for Medical Research, Lowy Cancer Research Centre, UNSW Sydney, Kensington, NSW, Australia; Institute for Glyco-core Research, Nagoya, Japan; Department of Biochemistry, Nagoya University Graduate School of Medicine, Nagoya, Japan

**Keywords:** neuroblastoma, scRNA-seq, spontaneous regression, Th-MYCN

## Abstract

**Background:**

Understanding the factors that determine the spontaneous regression of pre-cancerous lesions is critical to advancing cancer prevention. Neuroblastoma, a pediatric cancer, undergoes spontaneous regression more frequently than other types of cancer.

**Methods:**

Here, we analyzed the transcriptomic features of spontaneous regression in pre-cancerous neuroblasts using Th-MYCN mice, an animal model that closely resembles human neuroblastoma. Single-cell transcriptomic analysis of ganglion tissues from Th-MYCN mice was conducted to elucidate the cellular and molecular underpinnings.

**Results:**

Trajectory analysis of pre-cancerous neuroblasts revealed a distinct subtype we designated as “uncommitted” cells, characterized by the expression of neuronal genes, indicative of a semi-differentiated state. Samples with predicted failed tumorigenesis had a greater proportion of these uncommitted cells, hinting at their association with spontaneous regression. In clinical specimens, heightened uncommitted gene expression corresponded with favorable neuroblastomas and an improved prognosis.

**Conclusion:**

Collectively, the identification of this novel neuroblastoma-related cell subtype and its transcriptomic signature not only enhances our understanding of spontaneous regression mechanisms but also holds potential for therapeutic advancements in treating neuroblastomas.

## Graphical Abstract

**Graphical Abstract:**
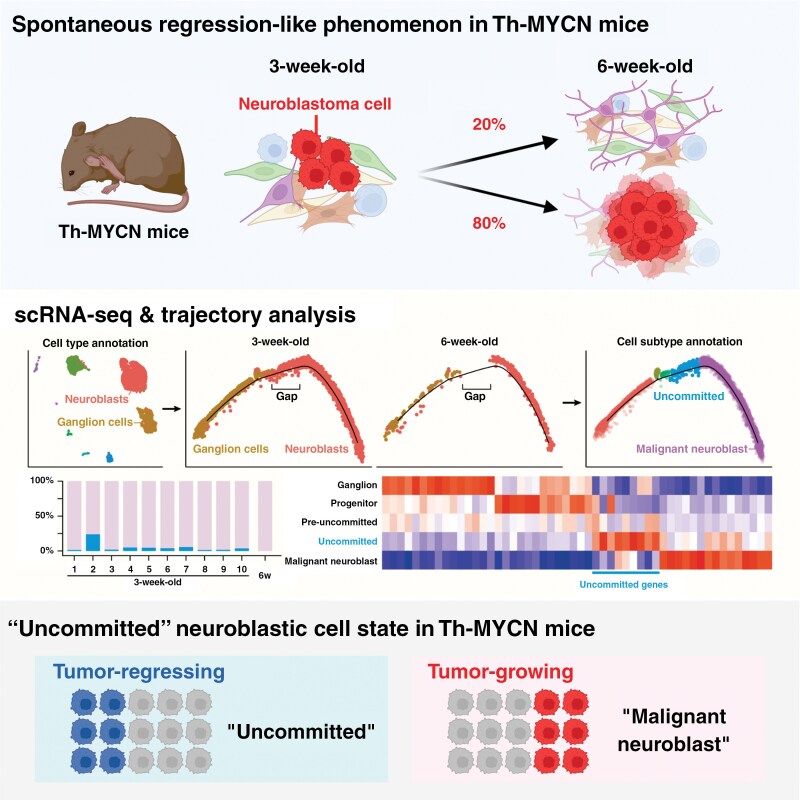

**Key Points**

- Single-cell transcriptomic analysis of pre-cancerous neuroblastoma in Th-MYCN mice
- A newly identified “uncommitted” subtype is associated with spontaneous regression
- Uncommitted gene signature is correlated with favorable human neuroblastoma

**Importance of the Study**

Understanding the enigmatic mechanisms of spontaneous cancer regression is critical in the progress toward cancer treatment and prevention. Neuroblastoma is a pediatric cancer undergoing spontaneous regression more frequently than other cancer types. Several plausible mechanisms of spontaneous regression have been proposed, but its etiology remains largely elusive. This study delves into the analysis of the transcriptomic features of spontaneous regression in pre-cancerous neuroblasts, utilizing the Th-MYCN mice model, which mirrors human neuroblastoma to a significant extent. This study unravels a previously unidentified neuroblastoma subtype—termed “uncommitted”—which surfaces early in pathogenesis. Our results spotlight this subtype’s association with spontaneous regression, thereby offering potential avenues for targeted cancer prevention strategies. Further, the growing emphasis on early cancer detection and the subsequent therapeutic interventions that might benefit from understanding the underlying mechanisms of spontaneous regression. The ability to recognize and potentially harness these mechanisms can revolutionize approaches to pediatric cancer management.

Neuroblastoma, a pediatric neuroendocrine tumor, originates from the developing adrenal gland and/or sympathetic ganglia. Neuroblastoma exhibits diverse clinical outcomes, varying from aggressive, untreatable tumor progression to spontaneous regression with or without minimal therapy.^1^ In 1964, Everson documented 130 cases of spontaneous cancer regression, most frequently observed in neuroblastoma.^2^ Brodeur also extensively reviewed the histories, biological features, and potential mechanisms of spontaneous regression of neuroblastoma.^3,4^ To date, 2 types of spontaneous regressions have been identified. The first type was detected through international mass urinary screening programs initially conducted in Japan,^5,6^ and subsequently in North America^7^ and Europe.^8^ These screenings identified neuroblastomas predominantly in infants aged 6 to 12 months without impacting overall mortality rates, leading us to classify this as pre-cancerous spontaneous regression. The second type was observed in patients with neuroblastoma exhibiting a localized tumor with dissemination limited to the liver and skin, with or without bone marrow involvement (designated stage 4S or MS).^9,10^ In either case, these tumors exhibited a favorable outcome, often regressing or differentiating with or without minimum therapy. Several plausible mechanisms of spontaneous regression have been proposed: (1) neurotrophin deprivation activating apoptosis through the TrkA-NGF pathway; (2) telomere shortening and apoptosis; (3) immune-mediated cell destruction; and (4) epigenetic modifications.^3,4^ However, its etiology remains largely elusive owing to a scarcity of pre-cancerous clinical samples.

The mechanisms underlying neuroblastoma genesis are well documented in animal models, especially in Th-MYCN mice, which is the most widely used neuroblastoma model. In these mice, the expression of the human *MYCN* gene is modulated by the rat tyrosine hydroxylase (Th) promoter.^11,12^ Genetic manipulation has influenced neuroblastoma development in Th-MYCN mice. For example, a heterozygous deletion of p53 in Th-MYCN mice has increased tumorigenesis and resistance to the anti-tumor drug cyclophosphamide.^13^ Similarly, mutant ALK (ALK^F1174L^ and ALK^R1279Q^) has significantly promoted tumorigenesis in Th-MYCN mice,^14,15^ while the deletion of caspase-8 has substantially heightened the metastatic potential of neuroblastoma into the bone marrow.^16^ Conversely, midkine knockout in Th-MYCN mice has inhibited neuroblastoma growth.^17^ Despite these insights, spontaneous regression of neuroblastoma has not been modeled so far owing to its enigmatic mechanisms.

In this study, we identified a phenomenon resembling pre-cancerous spontaneous regression in Th-MYCN mice by meticulously analyzing early tumorigenesis. Using single-cell RNA-sequencing, we delved into the cellular and molecular foundation of early stage neuroblastoma development, discovering a unique neuroblastoma cell subtype and its transcriptomic signature associated with this regression-like phenomenon in Th-MYCN mice.

## Methods

### Animals and Survival Analysis

WT and Th-MYCN mice, both derived from the 129^+Ter^/SvJcl mice background (CLEA Japan, Inc.). All animal experiments were approved by the Animal Care and Use Committee of Nagoya University Graduate School of Medicine (Nagoya, Japan). Th-MYCN mice were considered to have succumbed to tumors and were euthanized when they appeared unwell. Once tumors reached a palpable size, these mice were also euthanized. Kaplan–Meier survival analysis, accompanied by a log-rank (Mantel–Cox) test, was performed using GraphPad Prism software.

### Histological Assessment of SMG

SMG tissues were extracted along with their adjacent tissues and subsequently subjected to formalin fixation followed by paraffin embedding. For each mouse, serial paraffin sections of 5 μm thickness were prepared to capture the entirety of the SMG tissue. Staining was performed using hematoxylin 3G (8656, Sakura Finetek Japan) and eosin (8659, Sakura Finetek Japan). If the SMG displayed cells that histologically resembled neuroblastoma cells (typically such cells mostly consist of a nucleus and minimal cytoplasm, manifesting as small, dark purple cells), it was categorized as neuroblastoma (+). In the absence of such cells, it was classified as neuroblastoma (−).

### Tissue Dissection and Enzymatic Dissociation

Upon euthanization, the SMG of mice was meticulously dissected under the microscope. The SMG is anchored to the mesenteric artery and is proximal to the renal vein. Care was taken to detach the SMG using forceps without perturbing the adjacent blood vessels. Any attached fat was excised. The appearance of the dissected SMG was slightly white and transparent, as shown in Supplementary Figure S1A. This tissue was subsequently incubated in an equal ratio mix of collagenase IV (2.5 mg/mL, C5138, Sigma-Aldrich) and TrypLE Express Enzyme (12604013, Thermo Fisher Scientific) at 37 °C for 40 min. Afterward, 10 volumes of D-PBS (045-29795, FUJIFILM Wako Chemicals) containing 1% BSA (15260037, Thermo Fisher Scientific), DNase I (0.1 mg/mL, DN25, Sigma-Aldrich), and 10 mM MgCl_2_ were added, and the tissue was dissociated using a fire-polished Pasteur pipette until no substantial particles remained visible. The resultant cell suspension was centrifuged to obtain a cell pellet, which was then reconstituted in 1% BSA/D-PBS for subsequent analyses.

### Cytocentrifuge and Immunostaining for MYCN

A 200 μL cell suspension was centrifuged onto a CREST-coated slide (CRE-01, Matsunami Glass) using Cytospin 4 (Thermo Fisher Scientific) with a single cytofunnel (5991040, Epredia) at 800 g for 5 min. The attached cells were then immediately fixed with a 10% Formaldehyde Neutral Buffer Solution (37152-51, Nacalai Tesque) for 30 min at room temperature (RT). After washing thrice with D-PBS, slides were blocked using a solution containing 5% normal goat serum in 0.3% Triton X-100/D-PBS for 1 h at RT. They were then incubated with rabbit anti-MYCN (D1V2A) antibody (1:100, 84406, Cell Signaling Technology) in antibody diluent (1% BSA in 0.3% Triton X-100/D-PBS) overnight at 4 °C. This was followed by incubation with an Alexa Fluor 594-labeled goat anti-rabbit IgG secondary antibody (1:500, A-11012, Thermo Fisher Scientific) and DAPI solution (1:1000, D523, Dojindo) for 2 h at RT. Slides were mounted with Prolong Gold Antifade Mountant (P10144, Thermo Fisher Scientific) and imaged using an inverted microscope (BX41, EVIDENT) with the VS129 virtual slide system (EVIDENT). MYCN^+^/DAPI^+^ cells were quantified using Tissuemorph DP (Visiopharm).

### ScRNA-seq and Data Analysis

For scRNA-seq, libraries were prepared using Chromium Single Cell 3’ Reagent Kits v1 (10x Genomics) according to the user guide. Due to the limited number of cells in an SMG, the target cell recovery was set at 1,200 cells per sample. The sequenced reads were aligned to the mouse genome reference mm10 enriched with the human MYCN transgene sequence using the Cell Ranger analysis pipeline (10x Genomics). Droplets devoid of cells were filtered out using EmptyDrops, with a (FDR < 0.01).^18^ The resulting count matrices were further analyzed using the Seurat R package.^19^ Cell cycle scores for the S and G2M phases were calculated using published cell cycle gene sets.^20^ Cells with fewer than 200 detected genes, over 60% ribosomal gene content, or more than 10% mitochondrial gene content were excluded. Of the initial set, 11,982 cells were retained. These cells were normalized using the SCTransform function of Seurat, regressing out 2,000 variable genes with factors such as the number of genes, number of counts, mitochondrial and ribosomal gene percentages, and cell cycle scores for the S and G2M phases. Principal component analysis was performed, and component variances guided the determination of cutoffs for subsequent clustering and dimensionality reduction. In addition, the clustree R package was used to identify a stable clustering resolution parameter.^21^ Dimensionality reduction was performed via uniform manifold approximation and projection to visualize clustering results. Differentially expressed genes were identified using Seurat’s FindMarkers function, and cell types were annotated based on their specific markers. Gene Ontology analysis was conducted using the PANTHER overrepresentation test,^22^ while Enrichr, paired with the “ENCODE_and_ChEA_Consensus_TFs_from_ChIP-X” library, was used to ascertain potential upstream transcription factors/regulators of genes.^23^ Trajectory analysis was performed using the Monocle 2 R package.^24^ Pseudotime and trajectory calculations were performed based on previously reported gene sets found in ganglia or tumors from either WT or Th-MYCN mice.^25^ A gene expression pseudotime heatmap was plotted using the plot_genes_branched_heatmap function in the Monocle package. New cell subtypes were annotated based on cell positions on the trajectory, and differential gene expressions were determined.

### RNA-ISH and Quantification

We utilized the following RNAscope probes (Advanced Cell Diagnostics): Hs-MYCN (417501), Mm-Prph-C2 (400361-C2), and Mm-Ube2c-C3 (552191-C3).

#### RNA-ISH of tissue section

Mice were anesthetized and perfused with a sufficient volume of cold D-PBS containing heparin sodium (10 units/mL, 224122557, Mochida Pharmaceutical), followed by 4% paraformaldehyde (PFA)/PBS (163-20145, FUJIFILM Wako Chemicals). Subsequently, tissues were dissected, fixed in 4% PFA/PBS overnight at 4 °C, and subsequently replaced sequentially with 10%, 20%, and 30% sucrose (30403-55, Nacalai Tesque). They were then embedded in O.C.T. compound (4583, Sakura Finetek Japan) and frozen using a cold aluminum block and liquid nitrogen. We prepared 10-μm frozen sections on CREST-coated slide glasses with a cryostat (CM3050S, Leica Biosystems). RNA-ISH was performed using the RNAscope Multiplex Fluorescent Reagent Kit v2 with TSA Vivid Dyes (323270, Advanced Cell Diagnostics) as per the user manual. The pretreatment involved a 5-min target retrieval and a 5-min application of protease III. Post-RNAscope, we used the TrueBlack Plus Lipofuscin Autofluorescence Quencher (23014, biotium) to reduce autofluorescence, and slides were mounted with ProLong Gold Antifade Mountant with DAPI (P36931, Thermo Fisher Scientific).

#### RNA-ISH of cytocentrifuged cells

Cells were cytocentrifuged and fixed as previously described. These fixed cells were dehydrated using a 50%, 70%, and 100% ethanol gradient and stored at −20 °C. RNA-ISH was performed using the RNAscope Multiplex Fluorescent Reagent Kit (320850, Advanced Cell Diagnostics) following the user manual and pretreatment guide (320538-TN). After this procedure, slides were mounted with ProLong Gold Antifade Mountant with DAPI.

#### Data analysis

We acquired images using the super-resolution spinning disk confocal microscopy system (SpinSR10, EVIDENT). Signal spots per cell were quantified using the image analysis software HALO (Indica Labs) and its FISH-IF module.

### Immunofluorescence Staining

Frozen sections were prepared as described above. The following antibodies were used: MYCN (84406, CST), Prph (ab246502, abcam), and Ube2c (ab252940, abcam). The detailed protocol is provided in the Supplementary Methods.

### Sphere Culture Experiments

#### Sphere culture

Sphere culture was performed as previously reported.^26^ Briefly, spheres derived from 3-week-old Th-MYCN mice were cultured in a medium containing chick embryo extract, without retinoic acid. Spheres were then dissociated using the StemPro Accutase Cell Dissociation Reagent (A1110501, Thermo Fisher Scientific).

#### shRNAs and lentivirus preparation

For shRNA-mediated knockdown, the MISSION TRC2 pLKO.5-puro non-mammalian shRNA control plasmid DNA (SHC202, Sigma-Aldrich) and the MISSION pLKO.1-puro targeting specific genes (SHCLNG, Sigma-Aldrich) were procured (details in the Supplementary Methods). A lentivirus solution was prepared as previously reported^26^ (Supplementary Methods).

#### qPCR

Total RNA was isolated using the RNeasy Plus Mini Kit (74034, QIAGEN), and cDNAs were subsequently synthesized using the ReverTra Ace qPCR RT Master Mix with gDNA Remover (FSQ-301, TOYOBO). Quantitative PCR was conducted with the THUNDERBIRD SYBR qPCR Mix (QPS-201, TOYOBO) using either the Mx3000P or Mx3005P qPCR system (Agilent Technologies). The relative abundances of mRNA transcripts were determined through the 2^−ΔΔCT^ method, with normalization to Actb. Primer sequences are provided in the Supplementary Methods. *Sphere growth assay.* Sphere cells were seeded in a 96-well plate with white walls (655088, Greiner Bio-One). Sphere growth was assessed using the CellTiter-Glo 3D Cell Viability Assay (G9681, Promega), as per the manufacturer’s instructions. Relative sphere growth was determined using non-infected and non-puromycin-treated cells as the 100% reference.

#### Overexpression and retrovirus preparation

For overexpression of genes, Retro-X Tet-One Inducible Expression System (Puro) (634307, TaKaRa) was used, and coding regions of genes were cloned into pRetroX-TetOne-Puro vector. Retroviruses were produced using pVSV-G vector by GP2-293 packaging cells according to the manufacturer’s instructions. Sphere cells were infected with retroviruses, selected with puromycin at 0.2 μg/mL, and gene expression was induced by doxycycline at 5 μg/mL.

### Western Blotting

The following antibodies were used: Histone H3 (8173, CST), HMGB2 (14163, CST), DLK1 (ab210471, abcam), UBE2C (ab252940, abcam), Caspase-3 (14220, CST), and Cleaved caspase-3 (9664, CST). The detailed protocol is provided in the Supplementary Methods.

### Data Analysis of a Human Neuroblastoma Cohort

Data analyses of human neuroblastoma were conducted using the public dataset provided by Kocak.^27^ Gene expression signatures (uncommitted and malignant neuroblasts) were derived from the average *z*-scores of genes in each signature. Welch’s *t*-test was used to determine *P*-values. Kaplan–Meier analyses were employed for survival analysis, stratifying based on median expression to differentiate high and low expression. *P*-values were determined using log-rank tests.

## Results

### Spontaneous Regression-Like Phenomenon Occurs Before the Macroscopic Tumor Expansion of Neuroblastoma in Th-MYCN Mice

**Figure 1.**
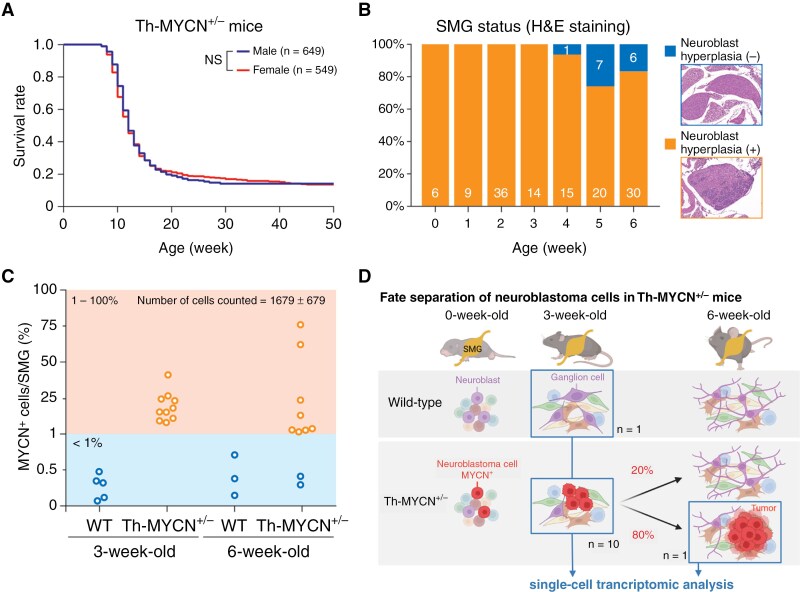
Spontaneous regression-like phenomenon in Th-MYCN mice. (A) Kaplan–Meier survival curves for male and female Th-MYCN hemizygote (Th-MYCN^+/−^) mice. NS, not significant. (B) Histological assessment of SMG in Th-MYCN^+/−^ mice using hematoxylin and eosin staining. Neuroblast hyperplasia (+) signifies the presence of pre-cancerous neuroblasts in the SMG. (C) Proportion of MYCN^+^ cells in an SMG determined by immunostaining of cytocentrifuged cells. Each plot represents an individual mouse-derived SMG. Values below 1% are comparable to WT mice and are deemed as negative SMG without MYCN^+^ cells. (D) Model illustrating the fate separation of neuroblastoma cells in Th-MYCN^+/−^ mice and the scRNA-seq experimental design. Created with BioRender.com.

Variation in tumor incidence among Th-MYCN mice is influenced by their background strains.^11^ In our study involving the backcrossed inbred 129^+Ter^/SvJcl mouse strain, all Th-MYCN homozygote (Th-MYCN^+/+^) mice succumbed to tumors (data not shown), while approximately 80% of Th-MYCN hemizygote (Th-MYCN^+/−^) mice succumbed to tumors, with the remaining 20% surviving without sex bias (Figure 1A). This raised questions about whether 80% of Th-MYCN^+/−^ mice experienced tumor initiation or if 20% potentially underwent spontaneous regression. To further understand, we investigated the early stage of tumorigenesis, around the weaning time ranging from 0 to 6 weeks of age. First, we assessed the superior mesenteric ganglia (SMG), the primary tumorigenesis origin in this strain—for the presence of pre-cancerous neuroblast cell clusters (neuroblast hyperplasia) using histological methods. Notably, all Th-MYCN^+/−^ mice displayed neuroblast hyperplasia up to 3 weeks of age (Figure 1B), higher than previously reported for different background strains.^28^ Yet, some mice lacked neuroblast hyperplasia from 4 to 6 weeks of age (Figure 1B). Subsequently, through immunostaining of a cytocentrifuged slide glass prepared following the dissection of a whole SMG tissue and enzymatic tissue dissociation (Supplementary Figure S1), we discerned that SMGs from 3-week-old Th-MYCN^+/−^ mice contained 1% to 50% MYCN^+^ cells, but some 6-week-old mice did not (Figure 1C). These results supported the premise that while all Th-MYCN^+/−^ mice displayed pre-cancerous neuroblasts for up to 3 weeks, their evolution into macroscopic tumors or their disappearance likely transpired earlier. The latter scenario possibly resulted from spontaneous regression in 20% of these mice (Figure 1D).

### Single-Cell Transcriptomic Analysis (scRNA-seq) of Ganglia From Th-MYCN^+/−^ Mice

**Figure 2.**
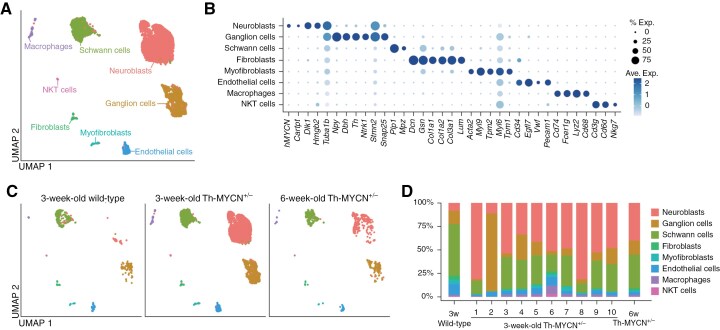
scRNA-seq of 8 cell types in Th-MYCN^+/−^ SMG. (A) UMAP with cell type annotations of 11,962 cells in SMG from 3-week-old WT (n = 1), 3-week-old Th-MYCN^+/−^ (n = 10), and 6-week-old Th-MYCN^+/−^ mice (n = 1). (B) Dot plot illustrating cell type-specific marker genes. (C) UMAP showing cell type annotations, color-coded by sample group. (D) Proportion of each identified cell type in each sample.

To investigate the cellular and molecular mechanisms underlying the fate determination of neuroblastoma cells in Th-MYCN^+/−^ mice, we performed scRNA-seq of SMG tissues from 3-week-old wild-type (WT, n = 1), 3-week-old Th-MYCN^+/−^ (n = 10), and 6-week-old Th-MYCN^+/−^ mice (n = 1) (Figure 1D). The 6-week-old Th-MYCN^+/−^ SMG was selected based on observed macroscopic tumors post-dissection, establishing it as a positive control for tumor growth. Post rigorous quality control, we obtained the transcriptome of 11,982 cells (Methods). Clustering analysis discerned 13 cell clusters including the human MYCN^+^ and MYC target signature^+^ cell cluster (Supplementary Figures 2A and 2B). These clusters were annotated into 8 cell types based on the differential expression of cell markers and signature scores: human MYCN^+^ neuroblasts (*Cartpt*, *Dlk1*),^29,30^ ganglion cells (*Npy*, *Dbh*, *Th*),^29,30^ Schwann cells (*Plp1*, *Mpz*),^29,30^ fibroblasts (*Dcn*, *Gsn*),^31^ myofibroblasts (*Acta2*, *Myl9*),^32^ endothelial cells (*Pecam1*, *Egfl7*),^33^ macrophages (*Cd74*, *Fcer1g*),^34,35^ and natural killer T (NKT) cells (*Cd3g*, *Cd3d*, *Nkg7*)^36^ (Figure 2A and 2B, Supplementary Figure S2C, and Supplementary Table S1). All cell types appeared in every sample group (Figure 2C). The “neuroblasts” cluster was even evident in SMG from 3-week-old WT mice, albeit in a low proportion. This suggests that undifferentiated/premature neuroblasts persist until this age during regular development or that they serve as tissue-resident stem cells.

A commonly discussed mechanism for spontaneous regression is anti-tumor immune responses.^3,4^ ScRNA-seq of established tumors from Th-MYCN mice has demonstrated infiltration by macrophages, T cells, B cells, dendritic cells, and other myeloid cells.^37^ However, our data showed that, while NKT cells and macrophages were present in Th-MYCN^+/−^ SMG, they appeared in low proportions and also in WT mice (Figure 2C and D). These results suggested that the mechanism for fate determination was unlikely due to specific cell types, such as immune cells, implying that immune-mediated cell killing was not the primary reason for spontaneous regression in Th-MYCN^+/−^ mice.

It is widely recognized that human neuroblastomas consist of 2 epigenetically regulated cell types: adrenergic/noradrenergic and mesenchymal/neural crest cell-like (NCC-like) cell types.^38,39^ We assessed signature scores for these cell-type-specific genes and found that MYCN^+^ neuroblasts predominantly expressed adrenergic/noradrenergic signatures but not mesenchymal/NCC-like signatures (Supplementary Figure S2D). Therefore, pre-cancerous neuroblasts identified at 3 weeks of age in Th-MYCN^+/−^ were primarily adrenergic/noradrenergic, suggesting that the previously defined epigenetically regulated cell states were not involved in fate determination at this stage.

### Trajectory Analysis Revealed a Unique Neuroblastoma Subtype Associated With Spontaneous Regression

**Figure 3.**
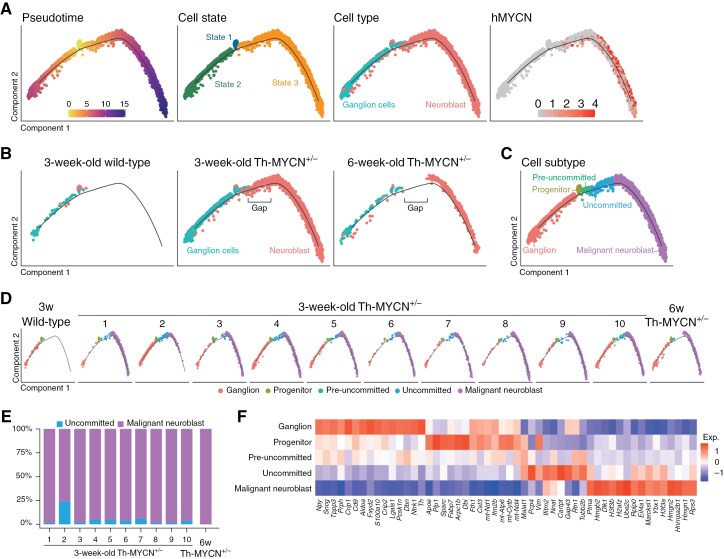
Trajectory analysis of ganglion cells and neuroblasts reveals a distinct neuroblastoma subtype. (A) Trajectory plots displaying pseudotime, cell state, cell type annotations from Figure 2, and human MYCN (hMYCN) expression via Monocle2. (B) Trajectory plots categorized by sample group with cell type annotations. (C) Cell subtype annotations derived from the trajectory plots in B. (D) Trajectory plots organized by sample with cell subtype annotations. (E) Proportion of uncommitted to malignant neuroblast populations per sample. (F) Heatmap depicting gene expression differences across cell subtypes.

We observed variations in the proportion of neuroblasts among 3-week-old Th-MYCN SMG samples. Notably, sample “#2” was different, containing fewer neuroblasts and predominantly ganglion cells (Figure 2D). Therefore, we focused on the 2 primary cell types (neuroblast and ganglion cells) in the following samples: 3-week-old WT SMG, 3-week-old Th-MYCN^+/−^ SMG, and 6-week-old Th-MYCN^+/−^ SMG. We performed trajectory analysis to discern subtypes within these cells. To construct this trajectory, we relied on a previously reported dataset derived from a microarray analysis of Th-MYCN ganglia. We then selected gene sets that were either upregulated (associated with tumor progression) or downregulated (associated with ganglion differentiation) during tumorigenesis.^25^ The resulting trajectory began with a small cluster of cells (state 1) and branched into 2 arms: the left arm, consisting of ganglion cells (state 2), and the right arm, which comprised MYCN^+^ neuroblasts (state 3) (Figure 3A and Supplementary Figure S3A). Cells in state 1, present in all sample groups from WT and Th-MYCN^+/−^, included both neuroblasts and ganglion cells. This suggests that these cells were possibly undifferentiated, non-malignant neuroblasts or progenitors of ganglion cells (Figure 3B).

A comparison of trajectories across the 3 sample groups revealed a distinct separation between the 3-week-old and 6-week-old Th-MYCN^+/−^ samples (Figure 3B). Therefore, we introduced a classification system with 5 cell subtypes: ganglion, progenitor, pre-uncommitted, uncommitted, and malignant neuroblast. This classification was based on the relative positions of cells within the trajectories of the 3 sample groups (Figure 3C). Notably, the proportion of the newly assigned “uncommitted” cell population varied among the 3-week-old Th-MYCN^+/−^ SMG samples (Figure 3D and E). Cells located at the extremity of the right arm exhibited elevated G2M and S phase scores (Supplementary Figure S3B). Moreover, these proliferating cells were absent in sample “#2” even though this sample exhibited a higher proportion of uncommitted cells (Figure 3D and E). Considering that 20% of Th-MYCN^+/−^ mice underwent spontaneous regression of neuroblast hyperplasia, we hypothesized that this uncommitted cell population was linked to this observed phenomenon.

Differentially expressed genes were identified for each cell subtype (Figure 3F and Supplementary Table S2). In the uncommitted population, only 9 genes were significantly overexpressed compared to other cell types (Supplementary Table S2). However, 46 genes showed significant overexpression when compared directly with the malignant neuroblast population (Supplementary Table S2). The PANTHER overrepresentation test^22^ revealed that these uncommitted genes (46 genes) were enriched in gene ontology terms associated with neuronal processes, such as “neuron death” (GO:0070997), “synaptic vesicle cycle” (GO:0099504), and “axon” (GO:0030424) (Supplementary Figure S4A). Using the Enrichr platform,^23^ we identified potential regulatory transcription factors for these uncommitted genes, including KLF4 and RUNX1 (Supplementary Figure S4B). Given the limited number of genes, confidence in the statistical significance of these findings was reserved. However, certain uncommitted-specific genes have been reported to be associated with neuroblastoma. For instance, MALAT1, a long non-coding RNA, plays a role in cell migration and invasion,^40^ as well as in neuronal differentiation.^41^ Both PCP4 and RTN1 are associated with neuroblastoma.^42,43^ Furthermore, KLF4 and RUNX1 are expressed in favorable neuroblastomas, and their expression is known to suppress neuroblastoma cell growth.^44,45^ Therefore, these genes might play pivotal roles in promoting the uncommitted population, potentially leading to pre-cancerous spontaneous regression.

In contrast, 125 genes were significantly overexpressed in malignant neuroblast populations relative to uncommitted ones (Supplementary Table S2). These malignant neuroblast genes (125 genes) were enriched in gene ontology terms associated with cell division (“regulation of chromosome segregation” [GO:0051983]), transcription (“regulation of mRNA processing” [GO:0050684]), and translation (“cytoplasmic translation” [GO:0002181]) (Supplementary Figure S4A). Enrichr analysis highlighted possible transcriptional regulators of these genes, including MYC (Supplementary Figure S4B). Given the elevated MYCN expression (Supplementary Table S2) and cell cycle scores (Supplementary Figure S3B), we inferred that the malignant neuroblast population was highly proliferative and represented a more malignant undifferentiated neuroblastoma cell type. This suggests they are primarily responsible for the eventual development of neuroblastoma tumors in Th-MYCN^+/−^ mice.

### Unique Neuroblastoma Subtype Emerges Exclusively During Early Tumorigenesis

**Figure 4.**
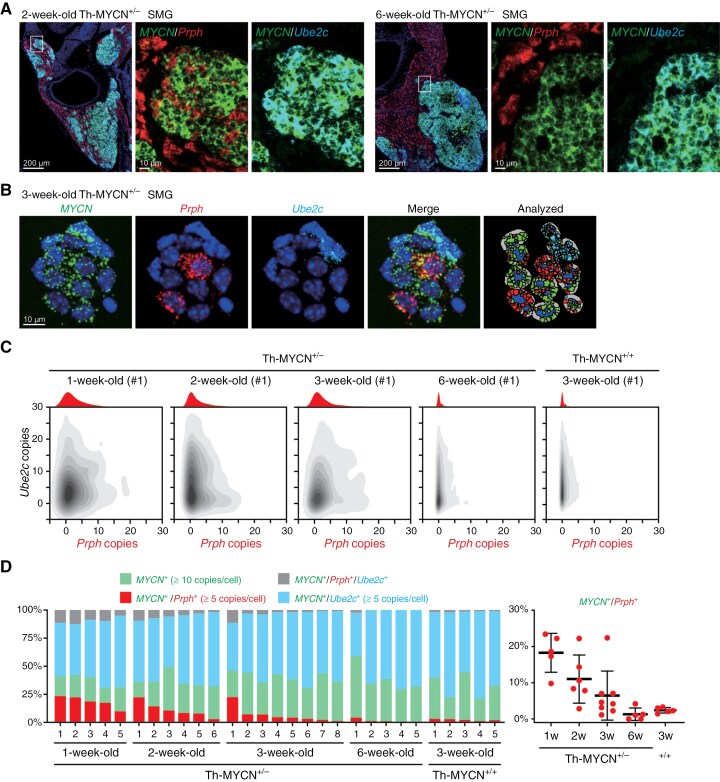
RNA-ISH revealed variations in the uncommitted population in Th-MYCN^+/−^ SMG. (A) RNA-ISH of MYCN, Prph (uncommitted marker), and Ube2c (malignant neuroblast marker) with DAPI (blue) on frozen sections of SMGs from 2-week-old and 6-week-old Th-MYCN^+/−^ mice. (B) RNA-ISH of MYCN, Prph, and Ube2c with DAPI (blue) on cytocentrifuged dissociated cells of SMGs from 3-week-old Th-MYCN mice. Signal spots per cell were analyzed and quantified using HALO. (C) Representative 2D density plots showing quantified *Prph* and *Ube2c* copies in MYCN^+^ cells. A histogram for MYCN^+^/Prph^+^ cells is displayed above the plot in red. (D) Distribution of MYCN^+^/Prph^+^ uncommitted cells in each sample (left), accompanied by a summary dot plot (right).

To validate the existence of an uncommitted population and investigate its dynamics during tumorigenesis, we performed RNA in situ hybridization (RNA-ISH). We selected human MYCN as a pan-universal neuroblastoma marker, with Prph and Ube2c signifying uncommitted and malignant neuroblast populations, respectively. While Prph was expressed in ganglion cells, its differential expression and the delta value of percentage cells expressed between the 2 populations made it a suitable choice (Supplementary Table S2). We initially performed RNA-ISH on frozen sections of SMG tissue from 2-week-old and 6-week-old Th-MYCN^+/−^ mice. In SMG samples from 2-week-old Th-MYCN^+/−^ mice, most MYCN^+^ neuroblastoma cells expressed Ube2c (Figure 4A). Notably, within these MYCN^+^ cell clusters, some cells appeared Prph^+^ and some seemed co-localized, making it challenging to ascertain whether any cells expressed both MYCN and Prph. In contrast, in SMG samples from 6-week-old Th-MYCN^+/−^ mice, most MYCN^+^ neuroblastoma cells were Ube2c^+^, expanding in tandem with MYCN^-^/Prph^+^ ganglion cells (Figure 4A). We confirmed the similar expression pattern of Prph by immunofluorescence staining in serial sections (Supplementary Figure S5A). Owing to the proximity of cells and challenges in single-cell quantification on tissue sections, we subsequently performed RNA-ISH on cytocentrifuged cells post-enzymatic tissue dissociation. We distinctly observed MYCN^+^/Prph^+^ uncommitted cells and MYCN^+^/Ube2c^+^ malignant neuroblast cells in SMG samples from 3-week-old Th-MYCN mice (Figure 4B). Signal spot quantification per cell (illustrated in “Analyzed” in Figure 4B) revealed MYCN^+^/Prph^+^ cells were present in SMG samples from 1-, 2-, and 3-week-old Th-MYCN^+/−^ mice. However, their presence waned in SMG samples from 6-week-old Th-MYCN^+/−^ mice and tumor tissue from 3-week-old Th-MYCN^+/+^ mice (Figure 4C). Although the proportion of MYCN^+^/Prph^+^ cells fluctuated between mice, a clear decline correlated with age (Figure 4D). Importantly, 1 SMG specimen from 3-week-old Th-MYCN^+/−^ mice (#1) exhibited a substantial number of MYCN^+^/Prph^+^ cells relative to other samples, implying that this mouse may undergo spontaneous regression. These results suggest that the uncommitted cell population emerges in the early tumorigenesis stages but diminishes with tumor growth. In other words, individuals who undergo malignant transformation or bypass spontaneous regression pathways tend to exhibit tumor growth.

### Functional and Clinical Relevance of the Th-MYCN^+/−^ Neuroblastoma Subtype

**Figure 5.**
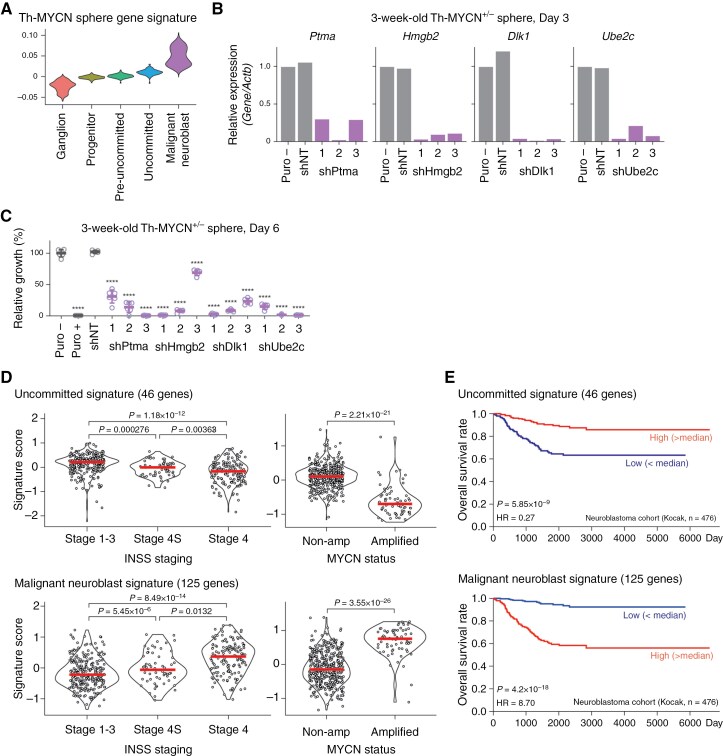
Functional and clinical relevance of the Th-MYCN^+/−^ neuroblastoma subtype. (A) Violin plots displaying Th-MYCN sphere gene signature scores from a previous study.^38^ (B, C) Relative mRNA expression of malignant neuroblast genes (B) and relative growth rates of 3-week-old Th-MYCN^+/−^ spheres infected with lentiviruses bearing shRNAs targeting these genes (C). Spheres infected with lentivirus were exposed to puromycin (0.2 μg/mL) for selection. “Puro” denotes spheres without infection and puromycin selection, while “shNT” indicates the non-targeting shRNA control. Data are presented as average ± SD. ns, not significant; **** *P* < .0001. (D) Violin plots showing signatures of uncommitted and malignant neuroblast genes in patients with neuroblastoma (Kocak cohort), categorized by INSS staging or MYCN status. (E) Kaplan–Meier survival curves representing the overall survival of patients with neuroblastoma, grouped by the median of signature scores for uncommitted and malignant neuroblast genes (high, red; low, blue). Associated log-rank *t*-test *P*-values are shown.

We previously determined the optimal spheroid culture conditions for pre-cancerous neuroblasts from Th-MYCN SMG and performed a microarray analysis to identify genes highly expressed in Th-MYCN spheres.^26^ The signature score of these Th-MYCN sphere genes was higher in the malignant neuroblast population compared to other subtypes (Figure 5A). This finding suggests that spheroid culture selectively enriches and expands the malignant neuroblast population in Th-MYCN mice, presenting a valuable tool for in vitro functional studies. We employed 3-week-old Th-MYCN^+/−^ spheres and lentivirus-mediated short hairpin RNAs (shRNAs) to target 4 genes (*Ptma*, *Hmgb2*, *Dlk1*, and *Ube2c*) that exhibited high expression in the malignant neuroblast population (Figure 3F and Supplementary Table S2). Each of the 3 different shRNAs targeting these genes significantly reduced the respective target gene mRNA and protein expressions by more than 50%, 3 days post-infection (Figure 5B and Supplementary Figure S6A). The knockdown of these malignant neuroblast genes may have led to the increased apoptosis as shown by cleaved caspase-3 (Supplementary Figure S6A), as well as potential increase in the expression of uncommitted genes to some extent (Supplementary Figure S7A). The knockdown of all these genes suppressed sphere formation 6 days post-infection (Figure 5C). Therefore, several genes enriched in the malignant neuroblast population (*Ptma*, *Hmgb2*, *Dlk1*, and *Ube2c*) play a crucial role in sustaining Th-MYCN^+/−^ derived spheres. This finding underscores that the acquisition of such malignant neuroblast gene expressions is pivotal for the full development of a macroscopic tumor in Th-MYCN^+/−^ mice.

Lastly, we investigated the clinical relevance of Th-MYCN^+/−^ neuroblastoma subtype signatures in human neuroblastoma utilizing the expression dataset from a large neuroblastoma cohort (Kocak cohort, GSE45547).^27^ Signature scores for uncommitted genes (46 genes) and malignant neuroblast genes (125 genes) were computed for each tumor. A heightened expression of the uncommitted gene signature was discerned in patients with favorable stages (stages 1-3 and stage 4S) and in those with non-amplified MYCN neuroblastoma (Figure 5D). In contrast, the malignant neuroblast gene signature was more prevalent in patients with stage 4 and those with amplified MYCN neuroblastoma (Figure 5D). The uncommitted gene signature was linked to a more favorable prognosis, whereas the malignant neuroblast gene signature indicated a poorer prognosis (Figure 5E). While the uncommitted gene signature was not specifically enriched in patients with stage 4S neuroblastoma, its elevated expression in neuroblastoma cases with favorable outcomes might hint that this specific neuroblastoma subtype holds the key to deciphering the relationship between neuroblastoma’s spontaneous regression and tumor progression.

## Discussion

Mechanisms underlying the spontaneous regression of neuroblastoma remain enigmatic, although some possible mechanisms have been proposed.^3,4^ In this study, we observed a pre-cancerous spontaneous regression-like phenomenon in Th-MYCN^+/−^ mice. To decipher the cellular and molecular underpinnings of this event, we utilized scRNA-seq and identified an “uncommitted” neuroblastoma subtype. This subtype exhibited a neuronal gene expression pattern, suggesting a slightly differentiated state. Although the exact sequence of neuroblastoma transformation is uncertain, our RNA-ISH assessment revealed that this unique uncommitted subtype emerges in early stages and diminishes with age. Notably, the highest uncommitted gene signature scores were observed in human neuroblastomas at stages 1 to 3 but were absent in stage 4S (Figure 5F). This suggests that Th-MYCN^+/−^ might not perfectly represent the biology of stage 4S neuroblastoma. The uncommitted gene signature likely resembles pre-cancerous regression observed in favorable neuroblastomas, such as those identified via mass urinary screening.^3,4^

A noteworthy aspect of our study pertains to the observed fate divergence within the same genetic backdrop (in our case, 129^+ter^/Svjcl mice). This discrepancy may be attributed to *MYCN* gene dosage. All Th-MYCN homozygote mice succumbed to tumors, underscoring the importance of *MYCN* gene expression in driving/promoting macroscopic tumor development. In the case of hemizygote mice, every SMG sample from 3-week-old Th-MYCN^+/−^ mice exhibited MYCN^+^ cells. However, scRNA-seq data indicated marginally reduced MYCN expression in uncommitted cells compared to malignant neuroblasts (Supplementary Table S2). Therefore, in uncommitted cells, intricate mechanisms might modulate MYCN expression at transcriptional levels or mRNA stabilization. Another possibility is that the acquisition of malignant characteristics requires additional genetic/epigenetic changes to fully promote tumor growth, which could be induced by MYCN dosage. Given that this fate separation event manifests during early development when normal developmental processes are ongoing, several environmental variables, including maternal factors during weaning, might play a role. Although we did not delve into the weaning effect, maternal factors could potentially influence neuroblastoma development, as previously reported.^46^ Single-cell RNA-sequencing of tumors derived from Th-MYCN mice in previous studies has demonstrated the presence of immune cells within the tumor tissue.^37^ These findings suggest that, in late-stage tumors, immune cells may constitute a significant component of the tumor microenvironment and potentially play a critical role in tumor progression or regulation. While our scRNA-seq data indicated the presence of NKT cells, they were scarce and even present in WT mice. Our results indicate that the number of immune cells is relatively low during the early stages of tumorigenesis; however, further studies are needed to determine whether immune cells are involved in tumor progression or spontaneous tumor regression. The potential effects of telomere shortening or nutrient deprivation via the NGF and TrkA pathways were not assessed in this study. However, the genes *Tert* (encoding telomerase), *Ngf*, and *Ntrk1* (encoding TrkA) were not differentially expressed between the uncommitted and malignant neuroblast populations (Supplementary Table S2). Therefore, established theories of spontaneous regression do not account for the observed phenomena in this animal model.

This study has several limitations. First, we were not able to identify critical factors, such as transcription factors that govern fate separation, specifically the induction of an uncommitted population or malignant neuroblastoma formation. In fact, we focused on the transcription factor Klf4, identified by Enrichr as potentially regulating uncommitted genes (Supplementary Figure S4B). RNA-ISH revealed MYCN^+^/Klf4^+^ cells, but they were scarce (less than 1%) (data not shown), making it challenging to ascertain Klf4 as the definitive regulator of uncommitted genes. Second, isolating the uncommitted population from Th-MYCN^+/−^ SMG tissue for functional experiments proved challenging. While a minor difference in gene expression between uncommitted and malignant neuroblast populations was noted, the absence of a distinguishing cell surface antigen for the isolation of cells using antibodies (Supplementary Table S2). Third, we attempted to ectopically express uncommitted genes in Th-MYCN^+/−^ derived spheres as a source of malignant neuroblasts (Supplementary Figure S8). We hypothesized that ectopic expression of uncommitted genes in Th-MYCN^+/−^ spheres would suppress sphere growth. However, such results were not observed. In fact, we were not able to overexpress these genes, possibly owing to the presence of potent gene suppression mechanisms in Th-MYCN spheres. We also acknowledge that sphere models may not fully capture the biology of intact ganglia in mice, as we have previously shown,^26^ particularly missing potential interactions with host cells and the involvement of multiple regulatory networks that may influence fate decisions in pre-malignant neuroblasts undergoing regression or progression to tumors. Collectively, this warrants further investigation into the functional assessment of the uncommitted population and future studies using emerging technologies such as spatial transcriptomics and lineage tracing may help clarify the molecular mechanisms underlying tumor progression or regression.

Nevertheless, our study revealed the previously unknown neuroblastoma uncommitted subtype, manifesting early in pathogenesis, and highlighted a novel mechanism underlying spontaneous neuroblastoma regression. To comprehensively understand the elusive mechanisms behind this spontaneous regression, further research using this model—alongside human neuroblastoma samples from the early stage of tumorigenesis (such as tumor tissues previously investigated by mass screening) or those from stage 4S patients are needed to fully understand the mechanisms underlying spontaneous regression.

## Supplementary material

Supplementary material is available online at *Neuro-Oncology* (https://academic.oup.com/neuro-oncology).

noaf129_Supplementary_Figure_S1

noaf129_Supplementary_Material

noaf129_Supplementary_Figure_S2

noaf129_Supplementary_Figure_S2D

noaf129_Supplementary_Table_S1

noaf129_Supplementary_Figure_S3

noaf129_Supplementary_Table_S2

noaf129_Supplementary_Figure_S4

noaf129_Supplementary_Figure_S5

noaf129_Supplementary_Figure_S6

noaf129_Supplementary_Figure_S7

noaf129_Supplementary_Figure_S8

## Data Availability

The single-cell RNA-sequencing data generated in this study are publicly available at Gene Expression Omnibus (GEO) at GSE247665.
