# Supplementary figures and images for: Trajectory analysis reveals an uncommitted neuroblastic state in MYCN-driven neuroblastoma development
Source: Neuro Oncol. 2025 Jun 24;27(10):2671–83. doi: 10.1093/neuonc/noaf129 (PMC12833547; doi:10.1093/neuonc/noaf129)

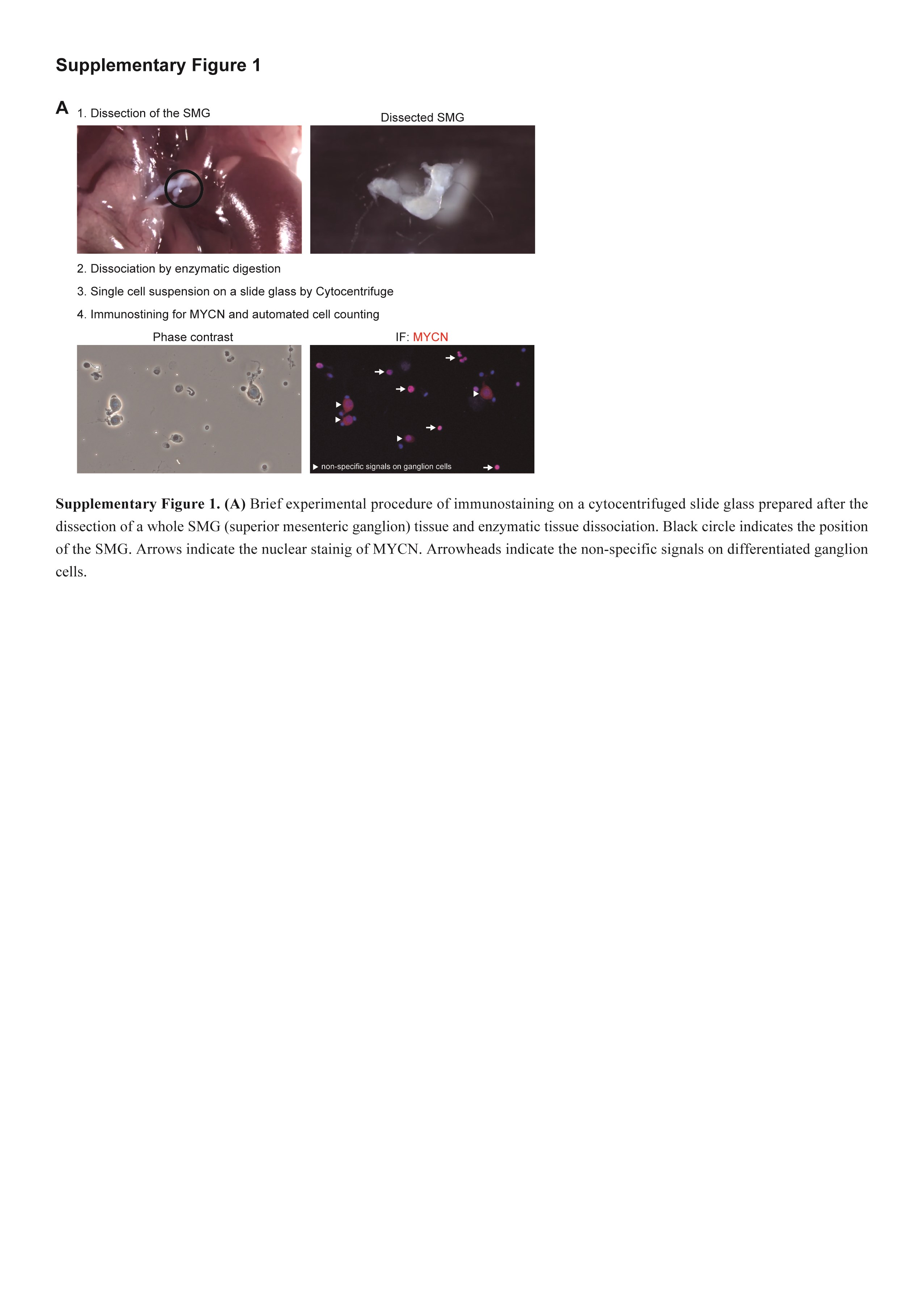

Supplement: noaf129_Supplementary_Figure_S1 [file noaf129_supplementary_figure_s1.jpeg]

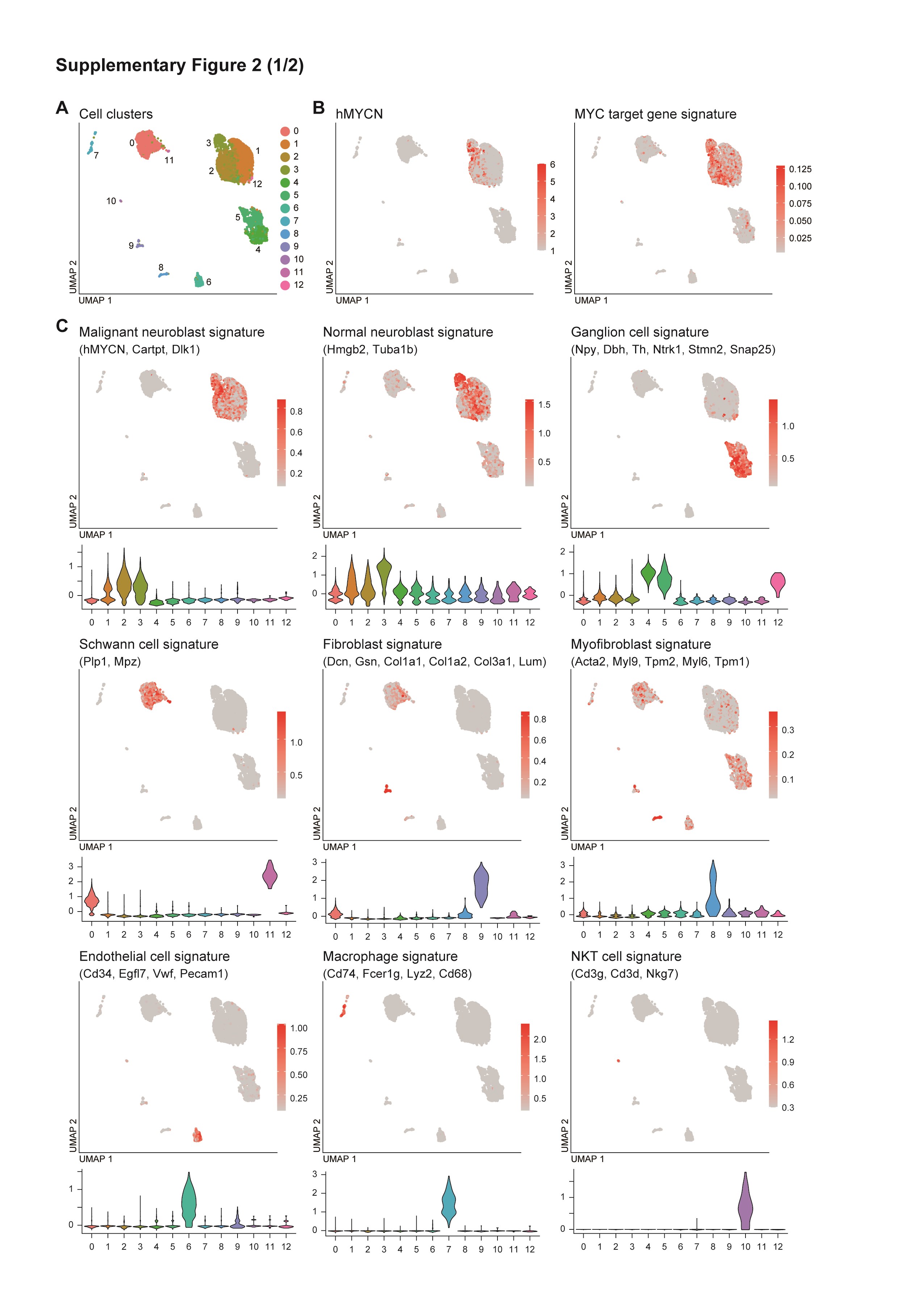

Supplement: noaf129_Supplementary_Figure_S2 [file noaf129_supplementary_figure_s2.jpeg]

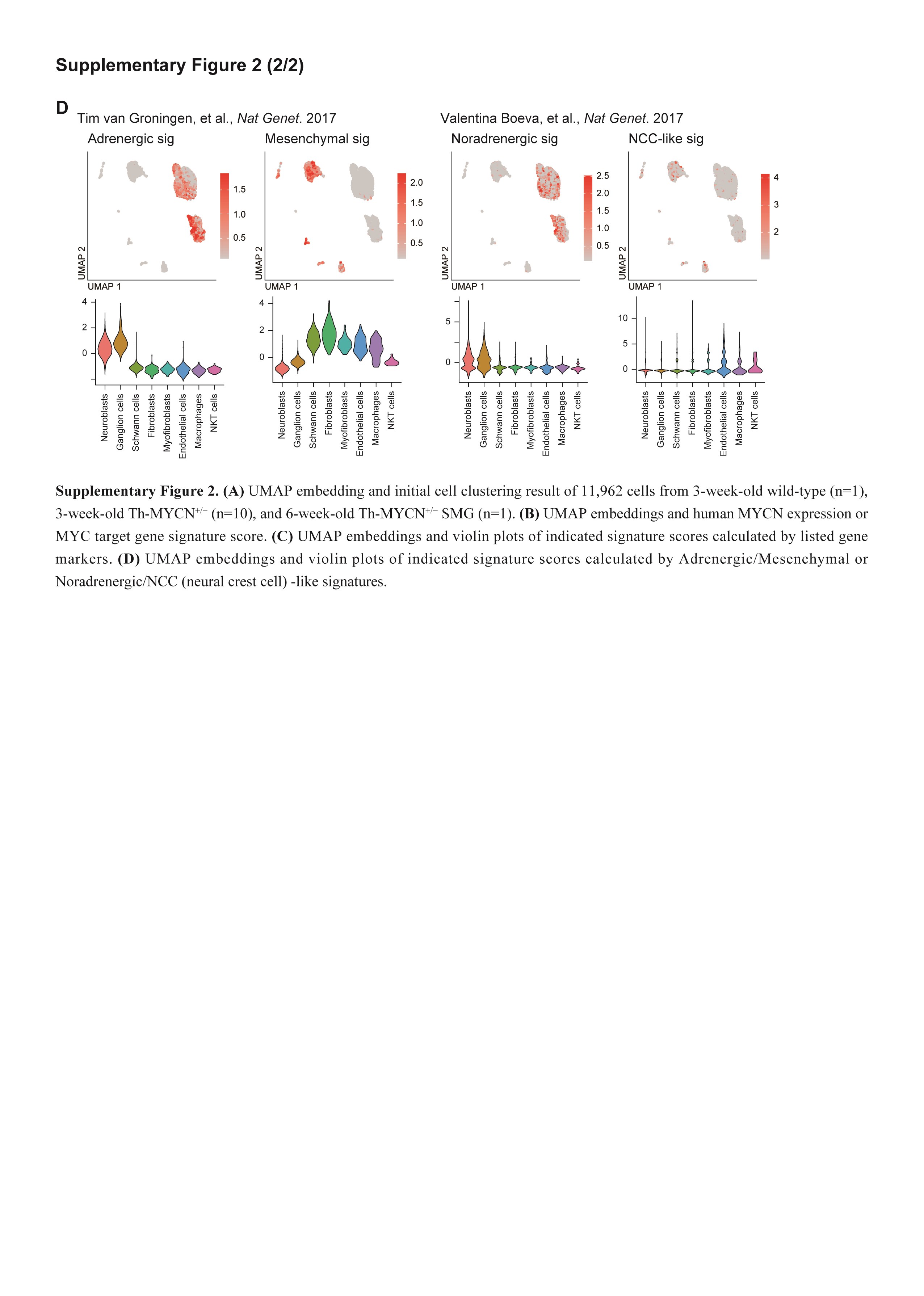

Supplement: noaf129_Supplementary_Figure_S2D [file noaf129_supplementary_figure_s2d.jpeg]

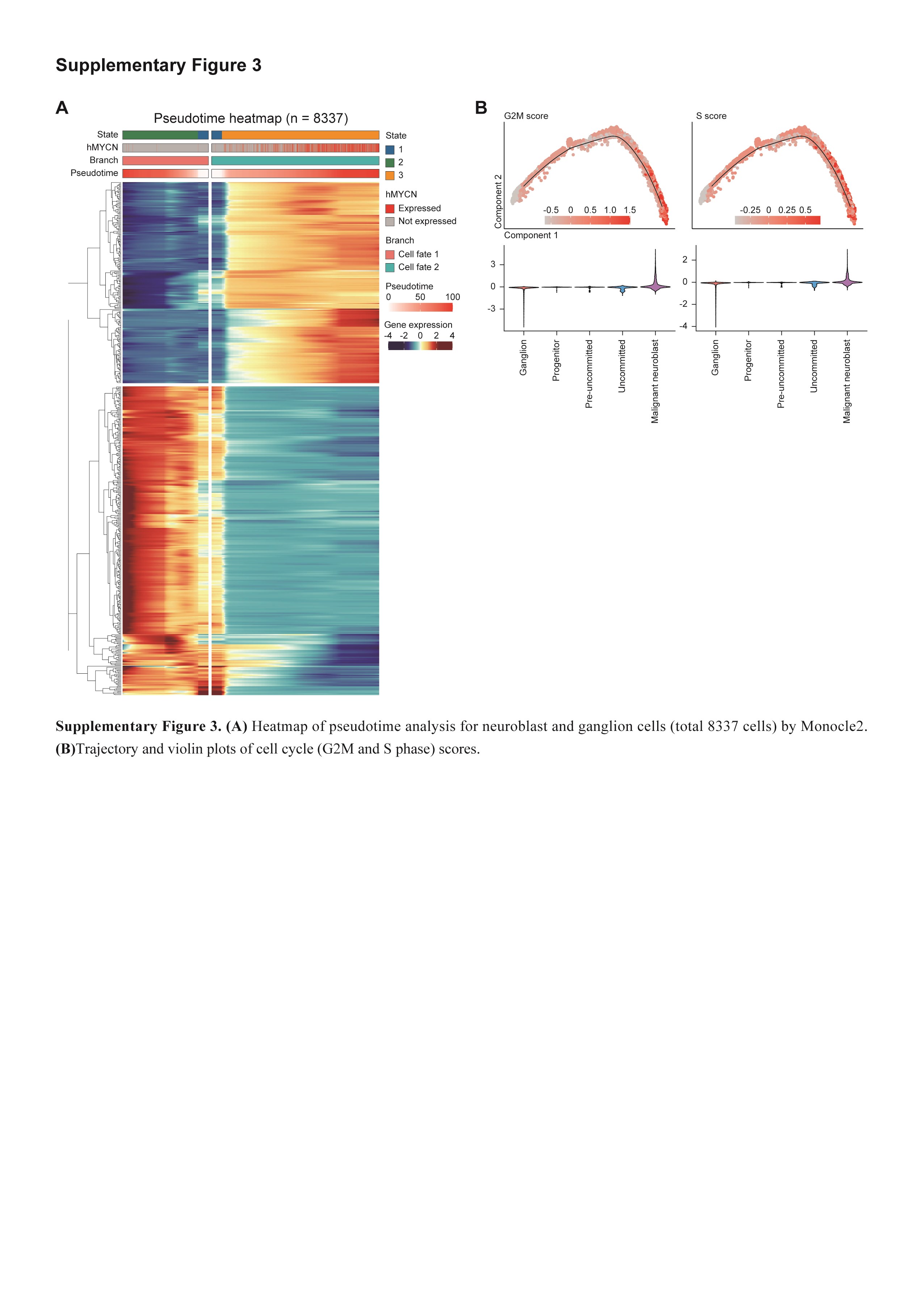

Supplement: noaf129_Supplementary_Figure_S3 [file noaf129_supplementary_figure_s3.jpeg]

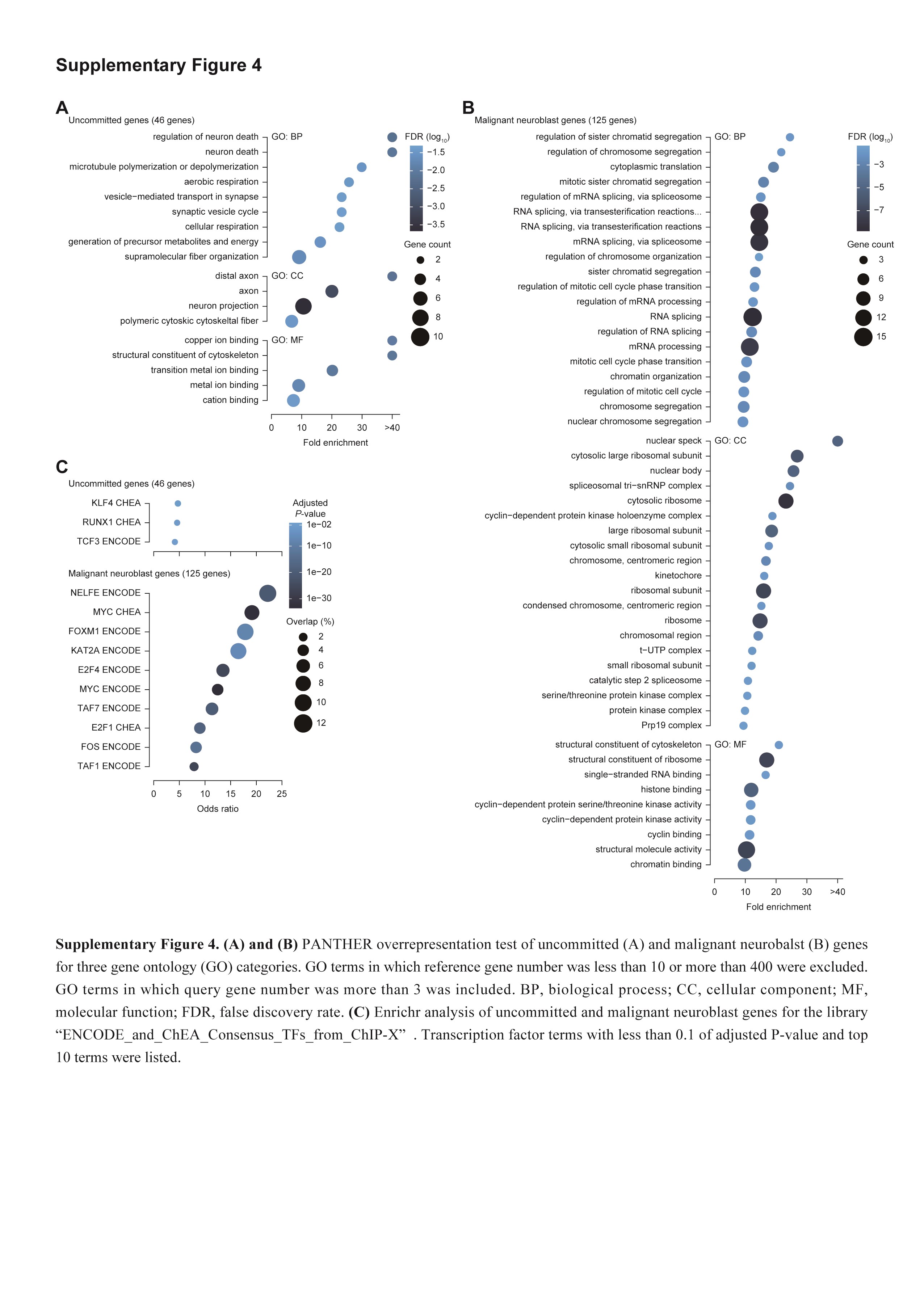

Supplement: noaf129_Supplementary_Figure_S4 [file noaf129_supplementary_figure_s4.jpeg]

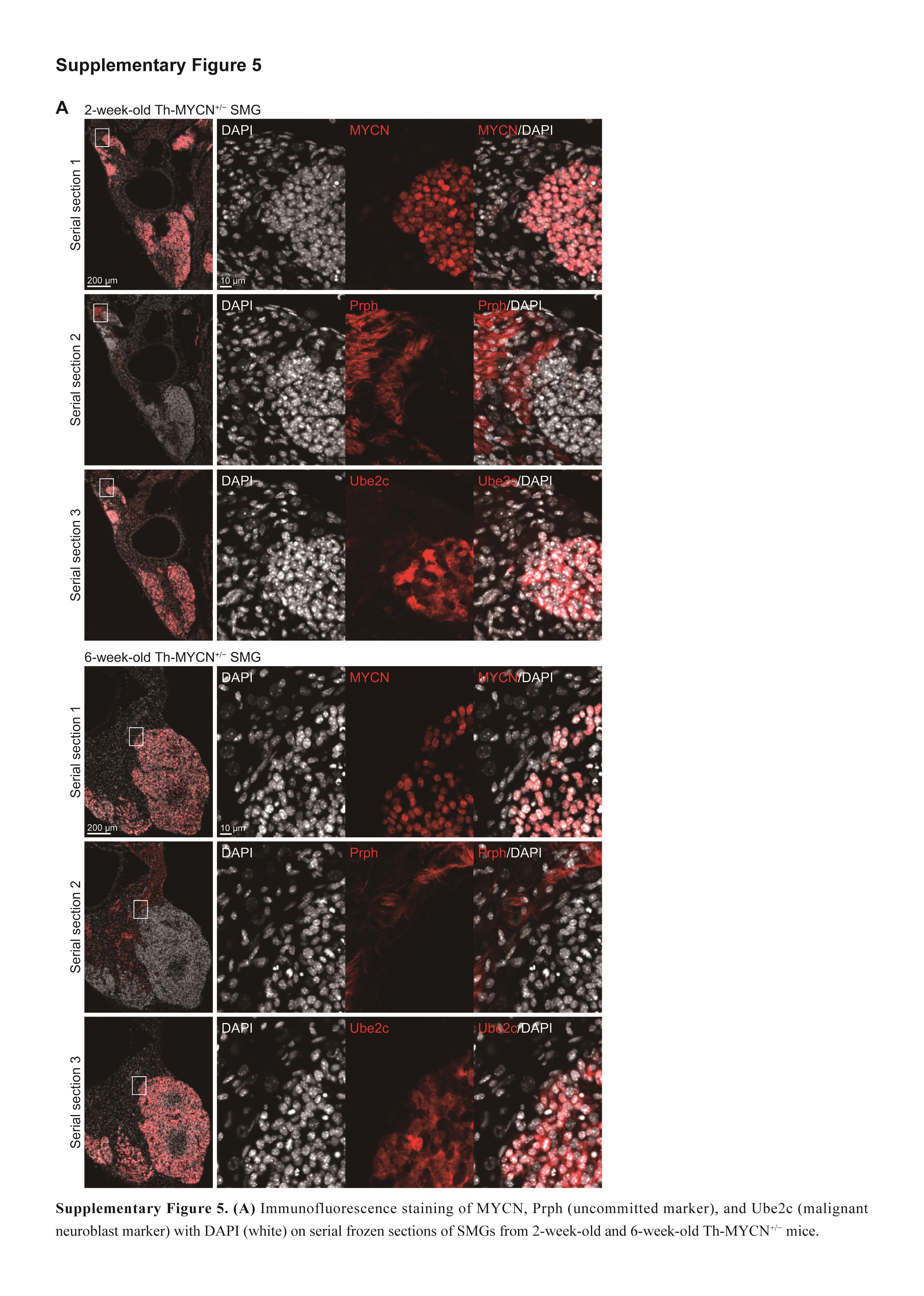

Supplement: noaf129_Supplementary_Figure_S5 [file noaf129_supplementary_figure_s5.jpeg]

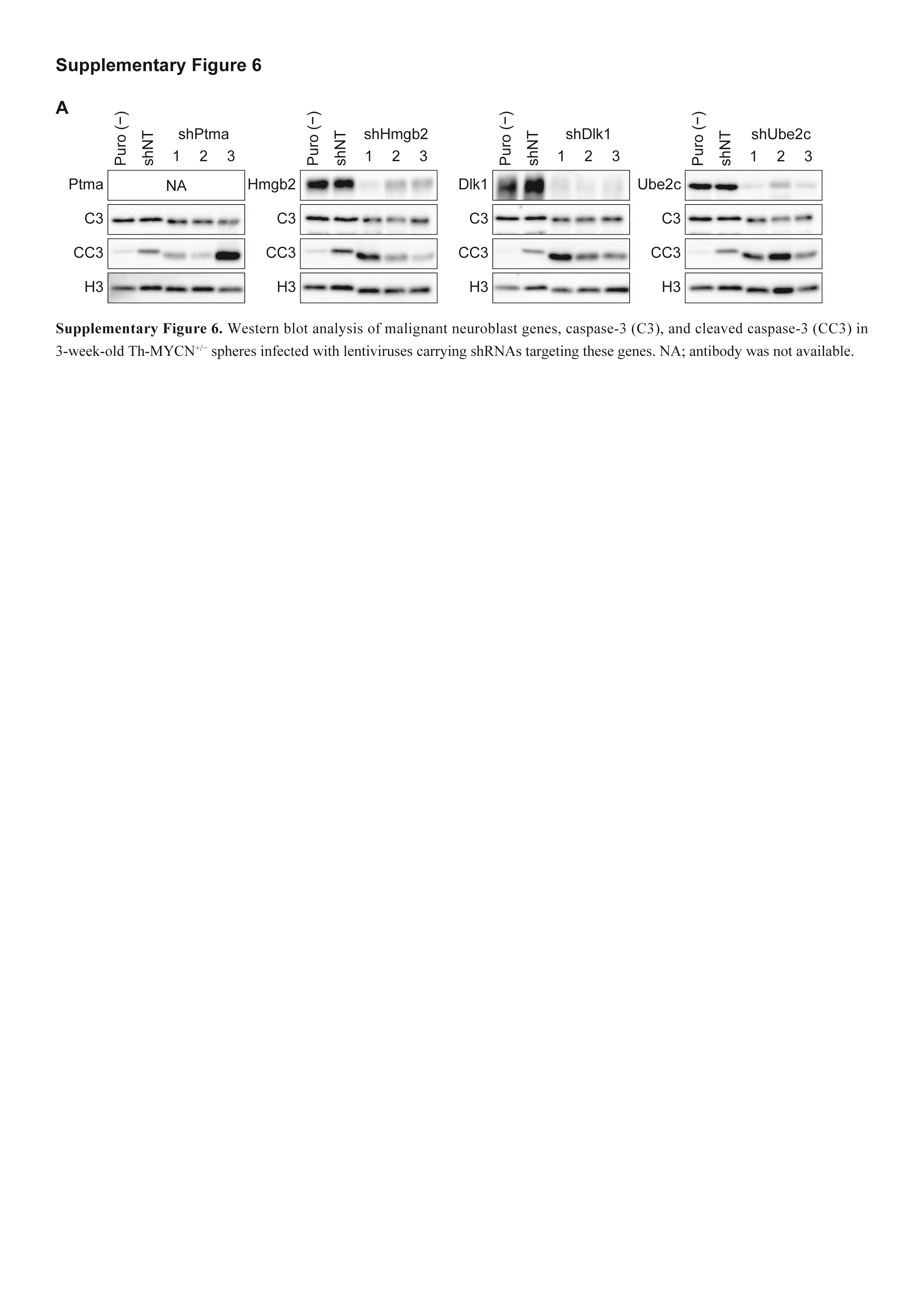

Supplement: noaf129_Supplementary_Figure_S6 [file noaf129_supplementary_figure_s6.jpeg]

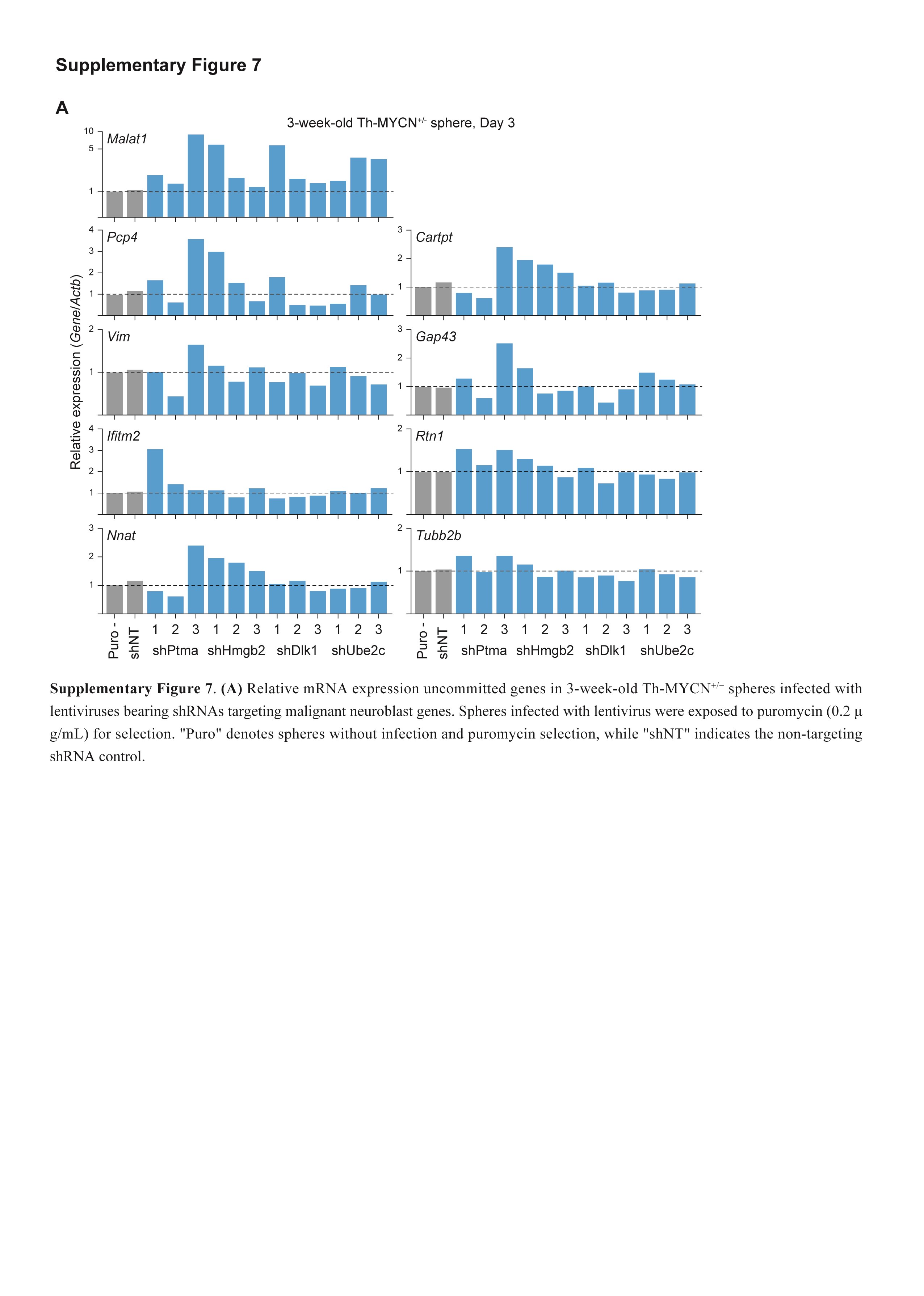

Supplement: noaf129_Supplementary_Figure_S7 [file noaf129_supplementary_figure_s7.jpeg]

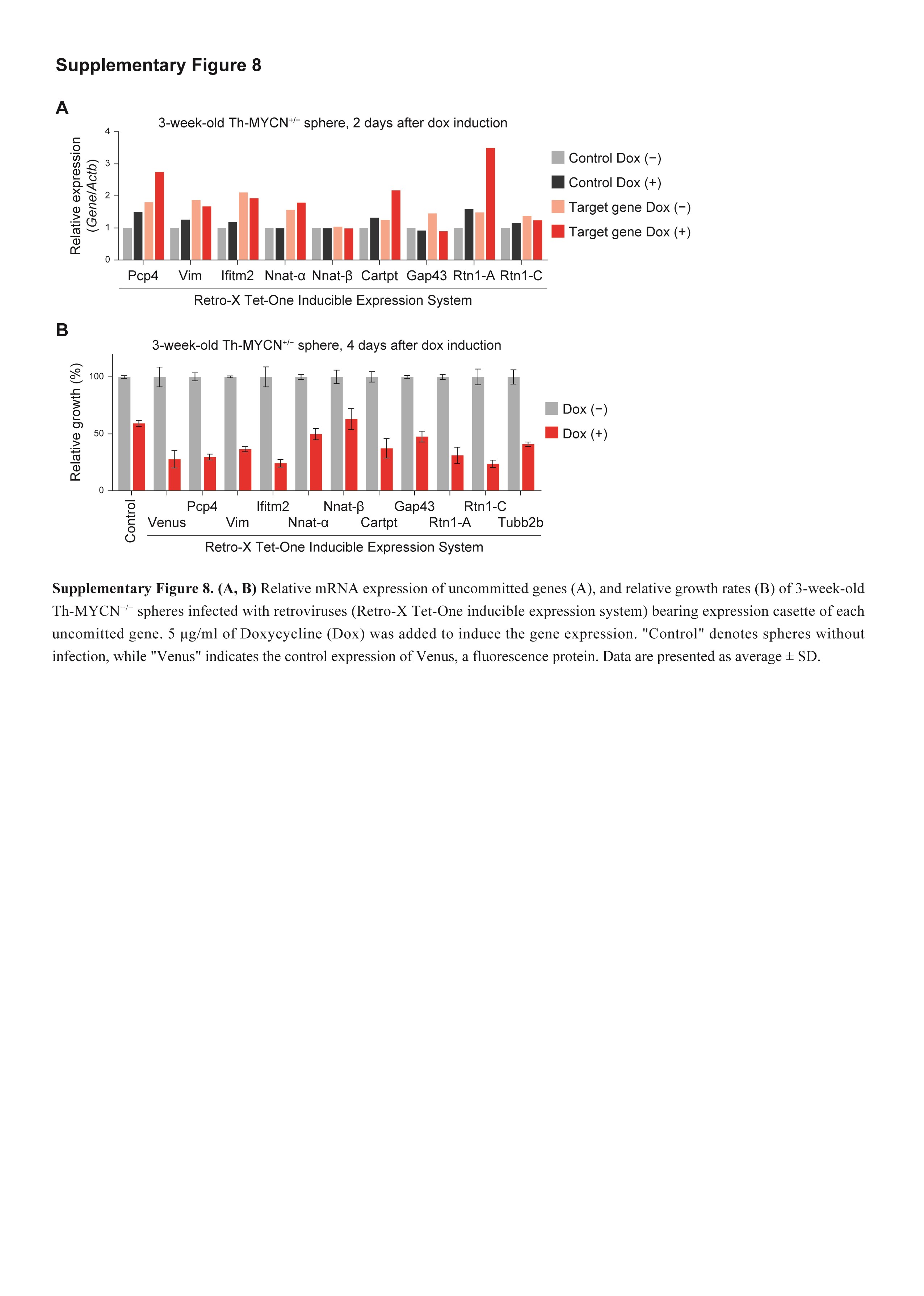

Supplement: noaf129_Supplementary_Figure_S8 [file noaf129_supplementary_figure_s8.jpeg]
