# Supplementary material for: Trajectory analysis reveals an uncommitted neuroblastic state in MYCN-driven neuroblastoma development
Source: Neuro Oncol. 2025 Jun 24;27(10):2671–83. doi: 10.1093/neuonc/noaf129 (PMC12833547; doi:10.1093/neuonc/noaf129)
Supplement: noaf129_Supplementary_Material [file noaf129_supplementary_material.docx]

**Supplementary Methods**

**Immunofluorescence staining**

Frozen sections were prepared as described above. The slides were immersed in D-PBS, blocked with blocking solution (5% normal goat serum in 0.3% Triton X-100/D-PBS), and incubated overnight with primary antibodies; MYCN (84406, CST); Prph (ab246502, abcam); Ube2c (ab252940, abcam) in antibody diluent (1% BSA, 0.3% Triton X-100/D-PBS). The slides were then incubated with secondary Alexa Fluor 647-labelled anti-Rabbit IgG (A32733, Invitrogen) with 4',6-Diamidino-2-phenylindole (DAPI) (D523, Dojindo) in antibody diluent. The slides were mounted with Prolong Gold Antifade Mountant (P36930, Invitrogen), and SpinSR10 was used to capture images.

**shRNA clone IDs and preparation of lentivirus**

The RNAi Consortium Clone IDs included: shPtma_#1, TRCN0000125363; shPtma_#2, TRCN0000417585; shPtma_#3, TRCN0000434086; shHmgb2_#1, TRCN0000301407; shHmgb2_#2, TRCN0000304268; shHmgb2_#3, TRCN0000304269; shDlk1_#1, TRCN0000334812; shDlk1_#2, TRCN0000334895; shDlk1_#3, TRCN0000363827; shUbe2c_#1, TRCN0000241128; shUbe2c_#2, TRCN0000241130; and shUbe2c_#3, TRCN0000241131.

Each lentivirus plasmid, combined with the Lentiviral High Titer Packaging Mix (6194, Takara Bio), was transfected into Lenti-X 293T cells using the TransIT-Lenti Transfection Reagent (V6603, Takara Bio). After 48 h post-transfection, the lentivirus-containing medium was collected and concentrated using the PEG-it Virus Precipitation Solution (LV810A-1, System Biosciences). The functional lentivirus titer was then ascertained using Lenti-X 293T cells through puromycin selection (ant-pr-1, InvivoGen), establishing a multiplicity of infection at 10. For the selection of lentivirus-infected cells, the puromycin concentration was set to 1 μg/mL for Lenti-X 293T cells and 0.2 μg/mL for the spheres.

**qPCR primer sequence**

The following primer sets were used:

| Gene | Forward | Reverse |
| --- | --- | --- |
| Mouse *Actb* | CACTGTCGAGTCGCGTCC | CGCAGCGATATCGTCATCCAT |
| Mouse *Ptma* | CTCTCGCCAGAGTCCTCGAA | GGAGCTGGTATCCACTGCC |
| Mouse *Hmgb2* | GCTCGTTATGACAGGGAGATG | TTGCCCTTGGCACGGTATG |
| Mouse *Dlk1* | AGTGCGAAACCTGGGTGTC | GCCTCCTTGTTGAAAGTGGTCA |
| Mouse *Ube2c* | CTCCGCCTTCCCTGAGTCA | GGTGCGTTGTAAGGGTAGCC |
| Mouse *Malat1* | TCGGCCTTGTAGATTAAAACGAA | AACGGCCGTCAACTTAACCT |
| Mouse *Pcp4* | GTGAGAGACAAAGTGCCGGA | TCTCTGGTGCATCCATGTCG |
| Mouse *Vim* | CGGCTGCGAGAGAAATTGC | CCACTTTCCGTTCAAGGTCAAG |
| Mouse *Ifitm2* | TGGGCTTCGTTGCCTATGC | AGAATGGGGTGTTCTTTGTGC |
| Mouse *Nnat* | AGCAGCACCGACAATGATGA | GGTGCCTACGCCCATATCTC |
| Mouse *Cartpt* | TGACTGTCCCCGAGGAACTT | ATATGGGAACCGAAGGTGGC |
| Mouse *Gap43* | TGGTGTCAAGCCGGAAGATAA | GCTGGTGCATCACCCTTCT |
| Mouse *Rtn1* | ACTGCCGATTCCACCAAGATG | TCCCGCCAGTACAGAAGGT |
| Moues *Tubb2b* | AGGCGGAGAGCAACATGAAT | CTTGGCCTGGGGAACTCAAG |

**Western blotting**

Protein was extracted in cell lysis buffer (20 mM Tris-Cl (pH 7.5), 150 mM NaCl, 1 mM Na_2_EDTA, 1 mM EGTA, 1% Triton X-100) containing cOmplete protease inhibitor cocktail (4693124001, Roche). The proteins were heated for 5 minutes at 95°C in sample buffer (62.5 mM Tris-Cl (pH6.8), 2% SDS, 10% Glycerol, 100 mM DTT). Denatured proteins were subjected to electrophoresis, transferred to a nitrocellulose membrane, blocked with 5% skim milk/TBST, and incubated overnight with primary antibodies. The membranes were incubated with secondary HRP-labeled goat anti-rabbit IgG polyclonal antibody (115-035-006, Jackson ImmunoResearch) in Can Get Signal Solution 2 (NKB-301, TOYOBO). Chemiluminescence was developed using Immobilon Classico or Forte (WBLUC0500, WBLUF0500, Merck Millipore), and the signals were detected on an Amersham Imager 600 (GE Healthcare).
